# Integrating Pharmacodynamic Data to Prioritise Candidate Migraine Genes and Pathways Using a Novel Bioinformatics Workflow

**DOI:** 10.3390/jcm15187040

**Published:** 2026-09-11

**Authors:** Mark Stanworth, Thomas Peukert, Joanne Marley, Clare Puddifoot, Andrew McDowell, Elaine Murray, Shu-Dong Zhang

**Affiliations:** 1 Personalised Medicine Centre, School of Medicine, Ulster University, C-TRIC Building, Altnagelvin Hospital, Londonderry BT47 6SB, UKe.murray@ulster.ac.uk (E.M.); 2 Department of Neurology, Royal Victoria Hospital, Belfast HSC Trust, Belfast BT12 6BA, UK; thomas.peukert@belfasttrust.hscni.net; 3 School of Health Sciences, Ulster University, Belfast BT5 1AP, UK; j.marley@ulster.ac.uk; 4 School of Medicine, Ulster University, Londonderry BT48 7JL, UK; c.puddifoot@ulster.ac.uk; 5 School of Biomedical Sciences, Ulster University, Coleraine BT52 1SA, UK; a.mcdowell@ulster.ac.uk

**Keywords:** migraine, genes, CHRM3, CHRNA4, SCN2B, accessory nerve, tension-type headache

## Abstract

**Background/Objectives**: Migraine is a neurological disorder with a heterogeneous presentation; however, compounds perceived as effective by migraine sufferers are often underrepresented in research. This study adopts a novel exploratory approach to determine whether pharmacodynamic data from such compounds can be used to prioritise candidate migraine-related genes and biological pathways for future investigation, and to generate hypotheses regarding migraine pathophysiology. **Methods**: A structured internet search identified 181 compounds perceived as effective for migraine, of which 148 had at least one supportive report identified in the PubMed literature. Following DrugBank-based exclusions for insufficient interaction data, 137 compounds remained for analysis. A gene list was extrapolated from compound–protein interactions, followed by analyses identifying significantly enriched tissues and KEGG (Kyoto Encyclopedia of Genes and Genomes) pathways. Unique genes from enriched tissue-specific sublists were considered targets of interest, and those not previously directly associated with migraine in the databases and literature searches used were deemed candidate migraine target genes. **Results**: A total of 32 compounds affected the products of 21 targets of interest. *CHRM3*, *CHRNA4*, and *SCN2B* emerged as prioritised candidate genes that had not previously been directly associated with migraine in the databases examined. Enrichment analysis of the pharmacodynamically derived gene set identified tissue-enrichment signals involving skeletal muscle (q = 6.01 × 10^−5^), prefrontal cortex (q = 5.36 × 10^−3^), cerebellar peduncles (q = 1.28 × 10^−2^) and cerebellum (q = 4.79 × 10^−2^). Nine compounds interacting with established Familial Hemiplegic Migraine (FHM) genes may warrant future investigation. No conclusions regarding therapeutic efficacy are drawn from these data. **Conclusions**: This exploratory pipeline prioritised *CHRM3*, *CHRNA4*, and *SCN2B* as candidate genes for further investigation, alongside enriched tissues and pathways. These findings should be regarded as hypothesis-generating rather than evidence of causality or therapeutic utility. More broadly, variations of this prioritisation framework may prove useful for exploring other disorders.

## 1. Introduction

Migraine is a trigeminally mediated neurological disorder with variable presentation, including headache, muscle weakness, and brain fog. It is associated with an increased risk of comorbidities such as musculoskeletal dysfunction [1]. Global prevalence is estimated at approximately 14–15% [2].

The first migraine-specific drugs, known as triptans, were developed in the 1990s based on the assumption that dural vasodilation caused headache (‘Vascular Theory’). Triptans are serotonin 5HT1_B/D_ receptor agonists that promote vasoconstriction; however, this mechanism is associated with increased cardiovascular risk. Subsequent research demonstrated that the trigeminal nerve (cranial nerve V, CN V) innervates the dural vasculature (‘Trigeminovascular Theory’), leading to the development of the first-in-class ditan. Lasmiditan, marketed as Reyvow, is a selective serotonin 5-HT1_F_ receptor agonist localised primarily to the brain and CN V, and has demonstrated a reduced cardiovascular risk profile compared with triptans. CGRP monoclonal antibodies and receptor antagonists were developed alongside lasmiditan to limit CGRP activity. Calcitonin Gene Related Peptide (CGRP), expressed by trigeminal neurons, induces dural vasodilation and inflammation via mast cell activation, thereby increasing headache intensity.

Despite the identification of genetic contributors to migraine, including *CACNA1A* in 1996 [3], *ATP1A2* in 2003 [4], and *SCN1A* in 2005 [5], no migraine-specific therapies have been developed that directly target the protein products of these genes.

Clinical research typically investigates a single drug targeting a specific disease mechanism, while off-target interactions are reported with largely unpredictable consequences. However, a perceived positive effect on migraine has been reported for various drugs and other medicinal substances not specifically investigated for this condition. There is therefore potential to identify candidate target genes and generate hypotheses regarding migraine pathophysiology through bioinformatic analysis of the collective drug–protein interactions of these agents.

This study investigated whether gene lists and individual genes extrapolated from drug–protein interactions could be used to prioritise candidate migraine-related genes and biological pathways for future investigation; note, this was an exploratory computational study and not an evaluation of therapeutic efficacy, safety, or clinical suitability. A secondary objective was to identify compounds interacting with established Familial Hemiplegic Migraine genes as observations that may inform future experimental research. The term ‘migraine’ is used here to refer specifically to the headache, regardless of subtype, as headache is the primary cause of disability common to all but one migraine subtype.

## 2. Materials and Methods

The following web resources were accessed: Google.com, duckduckgo.com, Enrichr (https://maayanlab.cloud/Enrichr/) (accessed on 22 October 2024) [6,7,8], DrugBank (https://go.drugbank.com/) (v.5.1.12, accessed on 21 October 2024) [9], PubMed (https://pubmed.ncbi.nlm.nih.gov/) (accessed on 21 October 2024), and HUGO Gene Nomenclature Committee (https://www.genenames.org/) (accessed on 22 October 2024) [10], Open Targets (https://platform.opentargets.org/) (v.26.06, accessed on 17 July 2026) [11], DisGeNET (https://disgenet.com/) (v.26.2, accessed on 17 July 2026) [12], OMIM (https://www.omim.org/) (accessed on 17 July 2026) [13], and GWAS ATLAS (https://atlas.ctglab.nl/) (v.20191115, accessed on 17 July 2026) [14]. Microsoft Excel was used for data collation.

Enrichr was the primary tool used for gene list analysis. It utilises Fisher’s exact test or the hypergeometric test and corrects for multiple hypotheses using the Benjamini–Hochberg method, yielding an adjusted *p*-value (q). Statistical significance was determined at α = 0.05.

This study did not follow a traditional study design; rather, it involved mining open-source data followed by bioinformatics analysis to elucidate migraine candidate target genes and pathways. The study was conceived as a novel approach to uncovering biological insights that may be missed by traditional study designs. A subsequent literature review was conducted to hypothesise mechanisms underlying the findings.

### 2.1. Compound Identification

Compounds perceived as effective for migraine were identified through a review of websites appearing within the first five pages of search results on Google.com and DuckDuckGo.com, using Boolean combinations of the keyword “migraine” with the terms “therapy,” “therapies,” “therapeutic,” “drug,” “treatment,” and “efficacy.” These search engines utilise different indexing methods, thereby reducing bias arising from search engine optimisation variability. The threshold of five pages was selected to balance the inclusion of a sufficient number of agents to obtain meaningful pharmacodynamic data against practical considerations of relevance and time management. The search strategy was used to generate a broad, exploratory list of candidate compounds.

Compounds were standardised to their generic names; combination therapies were separated, and duplicates were removed. Each agent (*n* = 181) was searched in PubMed alongside the term ‘migraine’ using a title/abstract query to identify evidence of a positive effect. As many compounds are directly reported by migraineurs as effective through websites and forums, and these claims are rarely followed up by formal research, a minimal inclusion threshold of one positive report in PubMed was applied. Agents with no supporting results were excluded (*n* = 33) to remove the weakest claims. This threshold was intentionally permissive as the purpose of the study was exploratory hypothesis generation, giving weight to direct reports of migraineurs rather than clinical evidence synthesis. Levels of published evidence were recorded to provide broader context and are presented in Supplementary File S1. Evidence levels were categorised as follows: animal models/expert opinion (Level 6), case reports/series (Level 5), case–control studies (Level 4), cohort studies (Level 3), randomised controlled trials (Level 2), and systematic reviews/meta-analyses (Level 1).

**Figure 1:**
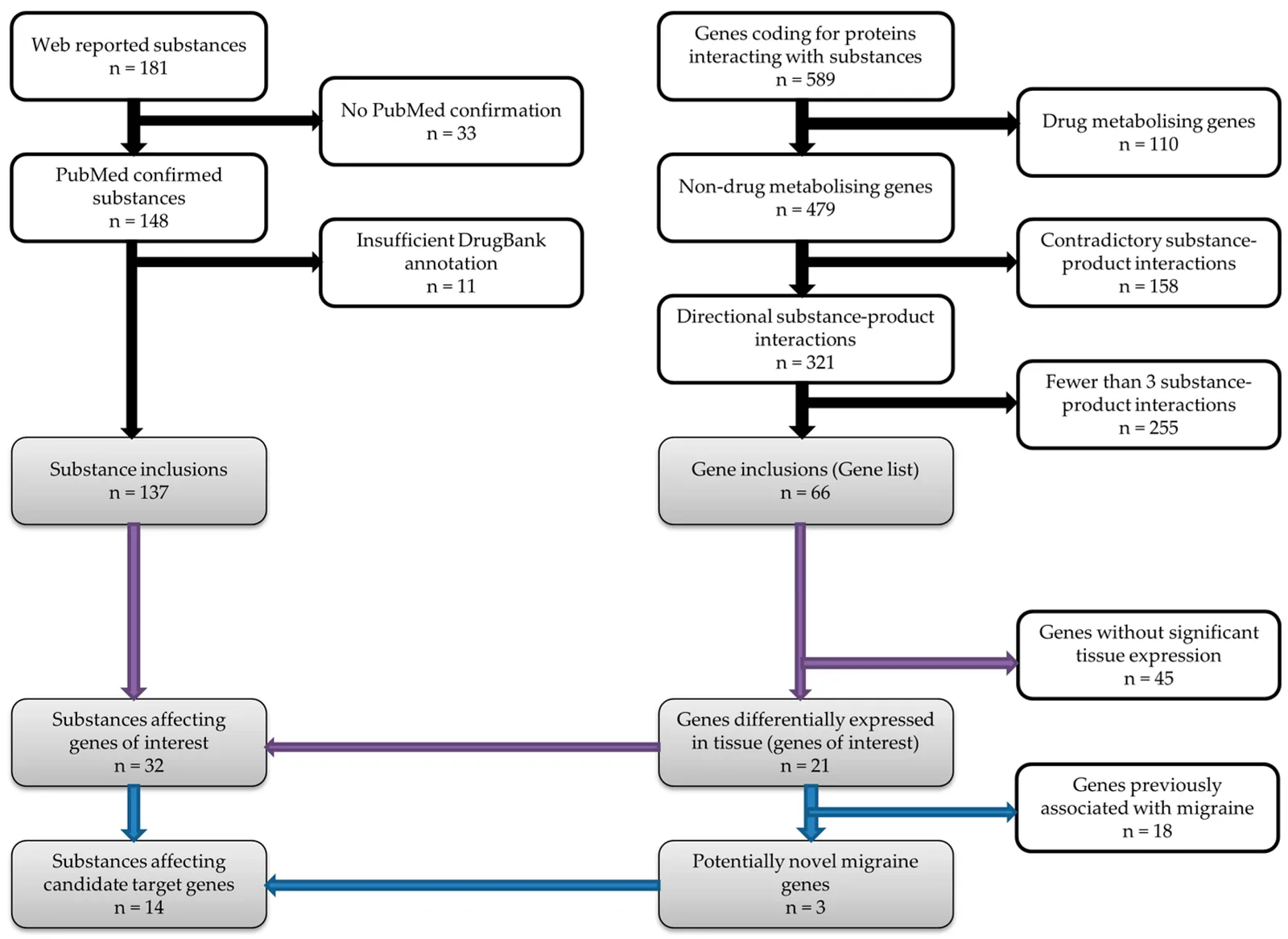
Exclusions from 181 compounds affecting 589 gene products. A total of 137 qualifying agents gave rise to a gene list of 66 genes. From this list, 21 unique genes were identified as differentially expressed in statistically significant tissue; 32 compounds interacted with their gene products. Three of these genes were candidate genes, which interact with 14 compounds.

DrugBank (v.5.1.12, accessed 21 October 2024) was consulted to identify drug–gene product interactions. Substances with insufficient interaction data, such as ‘stubs’ were excluded (*n* = 11), leaving 137 compounds. Agents affecting the products of migraine causal genes *CACNA1A*, *ATP1A2*, and *SCN1A* were noted for discussion regardless of interaction count. The exclusion flow is summarised in Figure 1.

### 2.2. Gene Extraction and Filtering

Human genes encoding the protein products that interact with the identified compounds were determined using DrugBank (v.5.1.12; accessed 21 October 2024). Where multiple entries existed, such as for lithium (e.g., lithium citrate, carbonate, hydroxide, cation, chloride, and succinate), the interactions for all entries were considered in the filtering process. Human genes encoding compound targets, enzymes, carriers, and transporters were recorded. Gene symbols were standardised using HUGO nomenclature, and duplicate genes were consolidated, yielding a total of 589 genes.

Some gene products function to remove xenobiotics from the body and therefore interact with many substances regardless of the intended drug target. To address potential confounding by drug metabolism, *ABC*, *CYP*, *GST*, *MAO*, *SLC*, *SLCO*, *SULT*, and *UGT* genes were excluded (*n* = 110). While these exclusions may have removed some true-positive migraine-related genes, they were intended to reduce bias towards drug metabolism pathways rather than migraine-related pathways. Consequently, these filtering criteria should be regarded as heuristic prioritisation rules rather than biologically validated decision thresholds. Alternative filtering strategies may identify different candidate genes. Genes whose products exhibited conflicting compound interactions (e.g., triptans act as agonists of HTR1_B_ and HTR1_D_, whereas ketamine acts as an antagonist) or binding interactions of unknown consequence were excluded (*n* = 158). These exclusions were based on the assumption that if target activation alleviates migraine, then target inactivation may promote it; therefore, excluding gene products affected by opposing actions was intended to reduce conflicting pharmacodynamic signals within the exploratory prioritisation framework. However, this approach may also exclude biologically relevant targets with context-dependent or bidirectional effects and therefore represents a methodological limitation. To reduce the influence of coincidental interactions, genes with fewer than three known interactions were excluded (*n* = 255), leaving 66 genes in the final gene list. DrugBank interaction categories (targets, enzymes, carriers, and transporters) were analysed collectively to maximise the available pharmacodynamic information. No weighting or distinction between these interaction classes was incorporated into the prioritisation process, as doing so would have excluded several classes due to insufficient numbers of genes to permit meaningful analyses. Additionally, separating genes into mutually exclusive functional categories would risk losing information due to pleiotropy. Prioritised genes should not be interpreted as equivalent biological targets but rather as candidate genes requiring subsequent category-specific evaluation in larger datasets.

Genes not represented in significantly enriched tissues or pathways (see below) were excluded (*n* = 45). A PubMed literature search was conducted using the term ‘migraine’ in combination with gene names and aliases, and genes previously associated with migraine were excluded (*n* = 16). DisGeNET, OMIM, Open Targets, and GWAS ATLAS were also interrogated, and genes associated with migraine were excluded (*n* = 2). This process left three candidate genes for discussion.

### 2.3. Tissues and Pathways Enrichment Analysis

Enrichment analysis was performed using Enrichr (https://maayanlab.cloud/Enrichr/ accessed on 22 October 2024) to identify enriched biological processes that could serve as a filter for candidate genes. The gene list was entered, and the Human Gene Atlas gene set library (GSE1133) was used to identify significantly enriched tissue terms. Genes overlapping with significant tissue terms were considered genes of interest and were isolated into sublists. Genes not included in these sublists (*n* = 45) were not considered further, although they may warrant future investigation. The tissue-enrichment step was used as a pragmatic prioritisation filter to reduce the candidate gene set for downstream investigation and manual review. It was not intended to establish that genes outside enriched tissues are biologically irrelevant to migraine. Rather, it represented a heuristic component of the exploratory workflow designed to focus subsequent analyses on genes contributing to statistically enriched biological signals.

The tissue sublists were entered into Enrichr, and the KEGG 2021 Human Pathways gene set library was used to identify enriched pathway terms. Most disorders are polygenic, and trigenic interactions tend to have a common biological process [15]. To improve the likelihood of identifying meaningful pathways, only significant results with a gene overlap of three or more were considered.

### 2.4. Familial Hemiplegic Migraine Analysis

Compounds interacting with the three established FHM causal genes (*CACNA1A*, *ATP1A2*, and *SCN1A*) were noted for future FHM research. A PubMed literature search using ’migraine’ AND the genes/pathways of interest was performed to contextualise the findings and identify candidate target genes.

## 3. Results

### 3.1. Compound Selection and Pharmacological Characteristics

A total of 181 compounds (Supplementary File S1, Table S1) were identified as being potentially effective for migraine, of which 137 met the criteria for inclusion. There were nine agents which interacted with products encoded by established FHM causal genes. Verapamil blocks the calcium channel encoded by the FHM1 causal gene, *CACNA1A*. No agents were found to interact with the product of the FHM2 causal gene, *ATP1A2*. Carbamazepine, cocaine, lamotrigine, oxcarbazepine, promethazine, topiramate, valproic acid, and zonisamide block the sodium channel encoded by the FHM3 causal gene, *SCN1A*.

### 3.2. Prioritise Genes for Candidate Migraine Targets

A total of 589 unique genes were identified, of which 66 met the criteria for inclusion (Table 1).

Of these, 21 unique genes were significantly enriched across four tissues and were considered targets of interest; 32 compounds interacted with these 21 genes (Supplementary File S2, Table S2). The causal genes *CACNA1A* and *ATP1A2* were excluded due to insufficient interactions. Serotonin receptor (*HTR*) genes were also excluded because of insufficient and/or conflicting interactions. The genes *CALCA*, *CALCB*, and *CALCRL*, which encode calcitonin gene-related peptide (CGRP) and its receptor, were initially included but were subsequently excluded because they did not contribute to any significantly enriched tissue. A total of 18 (86%) of the targets of interest had previously been reported to be directly or indirectly associated with migraine. No previous reports linking *CHRM3*, *CHRNA4*, or *SCN2B* to migraine were identified.

OMIM returned no associations between *CHRM3*, *CHRNA4*, *GABRB1*, *GRIN3A*, or *SCN2B* and migraine. There were no hits for *SCN2B*. DisGeNET returned no hits for *CHRM3*, *CHRNA4*, *GABRB1*, or *SCN2B*. The *GRIN3A* locus has been associated with migraine disorders. OpenTargets identified *CHRM3* mouse model phenotypes that included physical and behavioural features resembling some aspects of migraine, but no direct associations with migraine headache. *CHRNA4* was associated with neuropathic pain and inter-ictal attention impairment in migraineurs, but not directly with migraine headache. There was also clinical precedence for *GABRB1* through clinical trials involving topiramate and for *GRIN3A* through clinical trials of ketamine. GWAS ATLAS did not identify significant variants for *CHRM3*, *CHRNA4*, *GABRB1*, *GRIN3A*, or *SCN2B*.

### 3.3. Enriched Tissues and Biological Pathways

The gene list showed significant overlap with genes enriched in skeletal muscle, prefrontal cortex (PFC), cerebellum, and cerebellar peduncles (Table 2). Of the 21 unique overlapping genes, 32 compounds interacted with their protein products (Supplementary File S2, Table S2). Three of these genes had not previously been directly associated with migraine in the databases and literature searches used in this study and therefore remained prioritised candidate genes.

The skeletal muscle sublist returned 15 significant KEGG pathways, five of which were not related to skeletal muscle tissue (Table 3). Because several pathway enrichments were driven by only three or four overlapping genes, associated large odds ratios should be interpreted cautiously.

The prefrontal cortex sublist returned six significant KEGG pathways, one of which referred to unrelated tissue (Table 4).

No significant pathways were identified for the cerebellum or cerebellar peduncles. Of the 21 genes of interest, three met the criteria for candidate target genes. DrugBank recorded interactions between *CHRNA4* and the compounds amantadine, butalbital, and methadone; *CHRM3* and chlorpromazine, cyproheptadine, imipramine, methotrimeprazine, nortriptyline, pizotifen, promethazine, and tramadol; and *SCN2B* and cocaine, oxcarbazepine, and zonisamide.

### 3.4. Pharmacodynamic Mapping of Familial Hemiplegic Migraine Genes

Known pharmacodynamic annotations describing interactions between the compounds and the three established FHM causal genes (*CACNA1A*, *ATP1A2*, and *SCN1A*) were extracted from DrugBank to catalogue documented database-recorded interactions. For FHM1, verapamil was recorded as an inhibitor of *CACNA1A*-encoded calcium channels. No database interaction entries were identified for the product of the FHM2 causal gene (*ATP1A2*). Eight substances were annotated as inhibiting or blocking the sodium channels encoded by the FHM3 causal gene (*SCN1A*): carbamazepine, cocaine, lamotrigine, oxcarbazepine, promethazine, topiramate, valproic acid, and zonisamide. These database-recorded interactions are presented solely as a transparent mapping of existing pharmacodynamic literature. They should not be interpreted as evidence of therapeutic efficacy or clinical safety.

### 3.5. Sensitivity Analysis of Gene Interaction Thresholds

To assess the stability of the prioritisation workflow, a sensitivity analysis was performed by varying the minimum substance–product interaction threshold used during gene filtering. The primary analysis required a minimum of three interactions per gene, resulting in the list of 66 genes in Table 1, four significantly enriched tissues, and three prioritised candidate genes (CHRM3, CHRNA4, and SCN2B).

When the threshold was relaxed to two interactions, 91 genes were retained for enrichment analysis. Under this alternative threshold, skeletal muscle, prefrontal cortex, and cerebellar peduncles remained significantly enriched tissues (adjusted *p* < 0.05). The prioritised candidate genes CHRM3, CHRNA4, and SCN2B were all retained within the workflow.

When the threshold was increased to four interactions, 51 genes were retained for enrichment analysis. Under this more stringent threshold, skeletal muscle and prefrontal cortex remained significantly enriched tissues (adjusted *p* < 0.05). The candidate genes CHRM3 and CHRNA4 continued to be prioritised; however, SCN2B was no longer retained because it did not meet the stricter filtering criteria.

Overall, CHRM3 and CHRNA4 remained prioritised across all interaction thresholds examined, while SCN2B demonstrated greater sensitivity to increasingly restrictive filtering. These findings suggest that the principal observations of the workflow are reasonably stable to moderate variation in the minimum interaction threshold, although broader sensitivity analyses examining alternative filtering decisions and interaction classification strategies remain warranted.

## 4. Discussion

This exploratory study developed a novel bioinformatics pipeline utilising patient-reported efficacy and literature-supported drug–protein interactions to prioritise candidate migraine-related genes and pathways for further investigation, and to generate new hypotheses regarding migraine pathophysiology. Our computational analysis identified four enriched tissues, namely skeletal muscle, the prefrontal cortex, the cerebellum, and the cerebellar peduncle. It also prioritised 21 targets of interest according to our predefined filtering criteria. Among these, *CHRNA4*, *CHRM3*, and *SCN2B* remained prioritised because they had not previously been directly associated with migraine in the databases and literature searches examined. These genes should therefore be regarded as computationally prioritised candidates rather than validated migraine genes with a causal role in migraine pathophysiology. Together, the products of these three genes interact with 14 distinct compounds identified within our workflow, providing biological rationale for further investigation but not evidence of causal involvement in migraine. Although most genes of interest had previously been linked to migraine, our workflow illustrates how integrating pharmacodynamic interaction data with enrichment analyses can be used to prioritise testable hypotheses that complement, rather than replace, conventional genetic approaches.

The drug classes interacting with the greatest number of genes of interest were anticonvulsants (most notably topiramate), antihistamines, analgesics and benzodiazepines. These observations describe the composition of the pharmacodynamic dataset analysed in this study and should not be interpreted as evidence of therapeutic efficacy or superiority of any drug class in migraine. The prioritised gene set was dominated by sodium- and calcium-channel genes, consistent with the established role of ion-channel dysfunction in migraine pathology. However, whether this enrichment reflects underlying disease biology, biases within available pharmacodynamic databases, the compound selection process, or a combination of these factors cannot be determined from the present analysis. The high statistical significance of skeletal muscle in our analysis may reflect genuine peripheral contributions to migraine pathophysiology. Alternatively, because migraine and tension-type headache are frequently misdiagnosed as one another owing to overlapping symptoms [16], skeletal muscle enrichment may plausibly be a consequence of diagnostic overlap in patient-reported data.

### 4.1. CHRNA4 and the Neuromuscular Junction Hypothesis

The candidate gene *CHRNA4* encodes the nicotinic acetylcholine receptor alpha-4 subunit and was identified through the significantly enriched skeletal muscle sublist. *CHRNA4* variants have been associated with disorders affecting neuromuscular excitability, including sleep-related hyperkinetic epilepsy [17] and paroxysmal torticollis, highlighting its established role in cholinergic signalling and motor control. Within the skeletal muscle sublist, *CHRNA4* contributed to the statistical significance of several enriched pathways, including nicotine addiction, neuroactive ligand–receptor interactions, and cholinergic synapse pathways. These findings suggest that cholinergic signalling may warrant further consideration in migraine-related biology. It is possible that abnormal cholinergic signalling via *CHRNA4* receptors may contribute to migraine symptomology, potentially explaining why some patients experience symptoms resembling a mild cholinergic crisis (e.g., the ‘DUMBELLS’ symptom cluster). Smoking is associated with increased migraine risk [18], as well as muscle fatigue and atrophy [19]. Conversely, nicotine replacement therapy may exert antimigraine effects [20] and enhance muscle endurance [21], while nicotine withdrawal has been associated with migrainous monocular diplopia [22]. Because nicotine-related headaches frequently present with classic migrainous features, such as throbbing pain, photophobia, and phonophobia, diagnostic confusion is common and may have influenced previous clinical studies.

Furthermore, *CHRNA4* activity can downregulate sarcolemma connexin expression, thereby protecting against skeletal muscle atrophy [23], and mutations in mouse models have been shown to cause dystonia [24], highlighting its critical role in muscle control. These observations support the biological plausibility of *CHRNA4* involvement in neuromuscular function but do not establish a role in migraine pathophysiology. One hypothesis generated by the present analysis is that altered *CHRNA4* activity at the neuromuscular junctions may influence migraine susceptibility or symptom expression. Specifically, *CHRNA4* dysfunction at the neuromuscular junctions of the trapezius and sternocleidomastoid muscles could represent one possible mechanism warranting future investigation. These neck muscles are innervated by the spinal accessory nerve (CN XI), which converges with the trigeminal nerve (CN V) [25] within the trigeminocervical complex/nucleus [26]. Corticobulbar fibres of CN XI innervate the ipsilateral sternocleidomastoid and contralateral trapezius muscles, and trapezius muscle imbalance can result in referred pain and symptoms such as dizziness and blurred vision.

Aberrant *CHRNA4* activity could promote sustained sub-clinical muscle contraction or altered neck muscle tone, triggering nociceptive signalling that is clinically perceived as migraine headache. This mechanical link may explain why neck stiffness and pain are prominent ictal symptoms, and points to a shared molecular pathology with tension-type and cervicogenic headaches. Exercise, particularly neck endurance exercise, which primarily uses slow-twitch oxidative type I muscle fibres, can trigger migraine [27] and promote neck stiffness/pain [28,29], potentially through elevated calcitonin gene-related peptide (CGRP) [30]. Decreased trapezius muscle mass has been reported in migraineurs [31], possibly due to reduced size of fast-twitch glycolytic type IIB fibres [32]. While moderate- to high-intensity aerobic and resistance exercise has been shown to reduce migraine frequency, potentially through neck strengthening [33], glycolytic type IIB fibres produce high levels of hydrogen peroxide (H_2_O_2_) [34], which may contribute to muscle pain [35] and atrophy [36]. This mechanism could potentially help explain why certain forms of exercise trigger headache in susceptible individuals. Consequently, the relationship between *CHRNA4* mediated signalling and migraine biology may warrant further experimental investigation.

Collectively, these observations are compatible with, but do not establish, a role for *CHRNA4*-mediated neuromuscular dysfunction in migraine. Because *CHRNA4* was prioritised using an exploratory computational workflow based on pharmacodynamic interaction data, these findings should be regarded as hypothesis-generating rather than evidence of disease causality. Independent genetic, transcriptomic, and functional studies will be required to determine whether *CHRNA4* contributes directly to migraine susceptibility or symptomology.

### 4.2. CHRM3 and Cortical Excitability

Enriched within the prefrontal cortex sublist, *CHRM3* emerged as a prioritised candidate gene with biological functions compatible with central mechanisms implicated in migraine. Translocation of the *CHRM3* gene may result in vascular and neurological symptoms [37], while under-expression of *CHRM3* is associated with Major Depressive Disorder [38]. Furthermore, *CHRM3* receptors regulate rapid eye movement (REM) sleep [39], providing biological plausibility given the recognised association between sleep disturbance and migraine.

At the cellular level, *CHRM3* contributed to the significance of calcium signalling, neuroactive ligand-receptor interactions, and neurodegeneration pathways. CHRM3 activation drives G-protein-mediated intracellular calcium release, thereby promoting neuronal excitability. This increase in intracellular calcium mirrors the cellular excitability observed in gain-of-function *CACNA1A* mutations [3] that cause FHM1. Additionally, migraine has been associated with hypercalcemia [40], which may increase CGRP release. Transcranial Magnetic Stimulation (TMS) is thought to modulate calcium dynamics [41], and studies suggest that TMS targeting the dorsolateral PFC may be effective in migraine. Together, these observations support the biological plausibility of *CHRM3* involvement in pathways relevant to migraine, although they do not establish a causal role in disease pathology.

### 4.3. SCN2B and Trigeminal Pain Processing

The auxiliary sodium channel subunit *SCN2B* was significantly enriched in both the cerebellum and the cerebellar peduncles. While the pore-forming subunit *SCN1A* (Na_v_1.1) is a well-established FHM3 causal gene, auxiliary beta subunits such as *SCN2B* play critical roles in regulating channel gating kinetics, cell-surface expression, and localised nodal distribution in neurons.

*SCN2B* is highly expressed in the rat trigeminal ganglion [42] and has been directly linked to neuropathic and orofacial pain in murine models [43]. Hypernatremia in migraine has been recognised since 1951 [44], and network analyses have previously identified *SCN1B* as a significant contributor to neurological disorders including migraine [45]. These findings raise the possibility that *SCN2B* may contribute to migraine susceptibility through similar mechanisms. Collectively, these observations provide biological plausibility that *SCN2B* may influence trigeminal pain processing or cerebellar-mediated sensory gating.

### 4.4. Pharmacodynamic Mapping of FHM Genes

For FHM1 (*CACNA1A*), the calcium channel blocker verapamil was identified as a compound with a documented interaction with the gene product in DrugBank. No database-recorded interactions were identified for the product of FHM2 causal gene (*ATP1A2).* For FHM3 (*SCN1A*), eight compounds were annotated as inhibitors or blockers of the encoded sodium channel: carbamazepine, cocaine, lamotrigine, oxcarbazepine, promethazine, topiramate, valproic acid, and zonisamide. These observations represent a transparent summary of the pharmacodynamic interaction data identified through our workflow. They should not be interpreted as evidence of therapeutic efficacy, safety, prescribing suitability, or disease modification in FHM. Rather, they highlight compounds whose documented interactions overlap with established FHM genes and which may therefore be of interest for future mechanistic or experimental investigation.

### 4.5. Study Limitations

The findings in this study should be interpreted in the context of several methodological limitations. Firstly, our initial compound list relied on publicly available, patient-reported data identified through web searches, introducing the possibility of self-diagnosis errors and clinical misclassification. An international study reported correct migraine diagnosis rates of only 28% in primary care and 35% in secondary care settings [46]. Furthermore, the substantial overlap in symptoms between migraine and tension-type headache [16] may have influenced both compound inclusion and downstream analyses. Although a PubMed-based confirmation filter was applied to reduce the likelihood of including unsupported compounds, the possibility of false-positive therapeutic claims and inaccurate compound inclusion cannot be excluded.

Secondly, our drug-target mapping was constrained by the current scope and completeness of DrugBank annotations, and our results may partially reflect the biased landscape of existing pharmacological research rather than novel disease biology. While our stringent exclusion criteria were necessary to eliminate drug-metabolising confounders (such as *CYP* and *SLC* genes) and contradictory interactions, these strict filters may also have inadvertently excluded genuine therapeutic targets. For instance, our pipeline excluded the solute carrier gene *SLC1A3*, despite evidence linking its polymorphisms to reduced migraine risk [47]. Similarly, excluding genes with fewer than three interacting compounds, or removing those with opposing effects (e.g., agonist and antagonist activity at the same target) may have excluded biologically relevant genes exhibiting context-dependent or bidirectional pharmacological effects.

The use of tissue enrichment as a filtering criterion may also have excluded biologically relevant genes that did not contribute to significantly enriched tissue terms. Consequently, the final candidate-gene list should be interpreted as one possible prioritised gene set generated under the chosen workflow rather than a definitive representation of migraine-related biology. Alternative prioritisation strategies may yield different candidate genes. In addition, the tissue enrichment results should be interpreted cautiously because the input gene set was derived from DrugBank interactions rather than from an unbiased genomic dataset. DrugBank targets are disproportionately composed of pharmacologically tractable proteins, particularly receptors and ion channels. Consequently, enrichment against a whole-genome background may preferentially identify excitable tissues even in the absence of migraine specific biological signals. The significantly enriched tissues identified in this study should therefore be regarded as exploratory enrichment signals generated from this particular pharmacodynamically derived gene set rather than definitive evidence implicating those tissues in migraine pathophysiology. Future studies could evaluate the robustness of these findings using drug-target specific background sets or permutation based null models. Furthermore, some of the reported odds ratios were derived from small overlapping gene counts (for example, three or four genes), which can produce unstable estimates and should therefore be interpreted with caution.

Furthermore, the present workflow combined multiple DrugBank interaction categories (including targets, enzymes, carriers, and transporters) into a single prioritisation framework. Although this approach maximized the available pharmacodynamic information, these interaction types have distinct biological and pharmacological implications and were not analysed separately because some resulting gene sublists were too small to support meaningful enrichment analyses. Further studies should investigate whether category-specific analyses improve the robustness of candidate gene prioritization.

Finally, the prioritised gene set is dependent upon the filtering strategy employed. To assess the stability of the workflow, a limited sensitivity analysis was performed by varying the minimum substance–product interaction threshold. The results presented in Section 3.5 suggest that some aspects of the prioritisation framework are relatively stable to moderate variation in this parameter, while individual candidates may be sensitive to increasingly restrictive filtering. However, alternative methodological decisions, including the treatment of opposing pharmacodynamic interactions, exclusion criteria, inclusion of specific gene classes, and the handling of interaction categories, may also influence the final prioritised gene set. Consequently, broader sensitivity analyses and methodological evaluations will be required to establish the stability and generalisability of this prioritisation framework.

Overall, as an exploratory in silico study, *CHRNA4*, *CHRM3*, and *SCN2B* should be regarded as computationally prioritised candidate genes rather than validated migraine genes or therapeutic targets. Independent genetic, transcriptomic, and functional studies will be required to determine whether these genes contribute directly to migraine susceptibility, symptom expression, or underlying disease mechanisms.

## 5. Conclusions

In conclusion, this exploratory study presents a pharmacodynamic prioritisation framework for generating hypotheses regarding migraine biology. Application of this workflow prioritised *CHRNA4*, *CHRM3*, and *SCN2B* as candidate genes for further investigation rather than identifying validated migraine genes. Because these candidates were identified through heuristic computational filtering, they represent hypothesis-generating starting points rather than verified biological discoveries.

By utilising gene lists derived from pharmacodynamic data, our pipeline also identified four tissue-enrichment signals generated from the analysed pharmacodynamic gene set: skeletal muscle, the prefrontal cortex (PFC), the cerebellum, and the cerebellar peduncles. The interactions catalogued for these candidate genes provide a summary of recorded pharmacodynamics but do not constitute evidence of clinical efficacy, safety, or prescribing suitability. Ultimately, this exploratory workflow provides a computational framework for prioritising candidate genes for subsequent evaluation using genome-wide association studies, transcriptomics, and functional models.

## Figures and Tables

**Table 1:** Gene list (*n* = 66) derived from compounds meeting our defined criteria.

Gene List	Main Function
*CA1*, *CA2*	pH homeostasis. CO_2_ regulation
*CACNA1B*, *CACNA1C*, *CACNA1D*, *CACNA1E*, *CACNA1F*, *CACNA1G*, *CACNA1H*, *CACNA1I*, *CACNA1S*, *CACNB1*, *CACNB2*, *CACNB3*, *CACNB4*	Calcium signalling
*CALCA*, *CALCB* *CALCRL*	Neuropeptide signalling/receptor activity
*CALM1*	Intracellular calcium sensing and signal transduction
*CHRM2*, ***CHRM3***, *CHRM4*, *CHRM5* *CHRNA3*, ***CHRNA4***	Acetylcholine signalling
*GABRA4*, *GABRA6*, *GABRB1*, *GABRB2*, *GABRB3*, *GABRD*, *GABRE*, *GABRP*, *GABRQ*	GABA signalling
*GRIN1*, *GRIN2A*, *GRIN2B*, *GRIN2C*, *GRIN2D*, *GRIN3A*, *GRIN3B*	Glutamate signalling
*HRH1*, *HRH2*	Histamine signalling
*MTNR1B*	Insulin and calcium regulation
*NR1I2*, *NR3C1*	Transcription factor control, cytochrome P450 control
*OPRD1*	Opioid signalling
*PPARA*, *PPARG*	Metabolism regulation, adipogenesis
*PTGS1*, *PTGS2*	Prostaglandin production
*SCN1A* *, *SCN1B*, *SCN2A*, ***SCN2B***, *SCN3A*, *SCN3B*, *SCN4A*, *SCN4B*, *SCN5A*, *SCN7A*, *SCN8A*, *SCN9A*, *SCN10A*, *SCN11A*	Sodium signalling
*TRPV1*	Mediates pain (heat/chemical) sensitivity

Bold = candidate migraine target genes. * FHM3 causal gene.

**Table 2:** Tissue-specific sublists of genes enriched in the indicated tissues.

Skeletal Muscle	Prefrontal Cortex	Cerebellar Peduncles	Cerebellum
*n* = 10, OR = 8.08, *p* = 1.72 × 10^−6^, q = 6.01 × 10^−5^	*n* = 8, OR = 5.22, *p* = 3.06 × 10^−4^, q = 5.36 × 10^−3^	*n* = 4, OR = 9.61, *p* = 1.09 × 10^−3^, q = 1.28 × 10^−2^	*n* = 3, OR = 8.99, *p* = 5.47 × 10^−3^, q = 4.79 × 10^−2^
*CACNA1S*	*CACNA1I*	*CACNB4*	*CACNA1G*
*CACNB1*	*CACNB1*	*GABRA6*	*GABRD*
*CACNB4*	*CALM1*	*GABRD*	***SCN2B***
*CHRM2*	***CHRM3***	***SCN2B***	
*CHRM5*	*GABRB1*		
***CHRNA4***	*GRIN1*		
*GABRQ*	*SCN1A* *		
*GRIN2B*	*SCN1B*		
*GRIN3A*			
*SCN4A*			

Bold = candidate migraine target genes. * FHM3 causal gene.

**Table 3:** Significant pathways derived from the skeletal muscle gene sublist.

Term	*p*	q	OR	Genes
Nicotine addiction	2.85 × 10^−9^	1.05 × 10^−7^	369.52	*GABRQ*, *GRIN3A*, ***CHRNA4***, *GRIN2B*
Neuroactive ligand–receptor interaction	4.66 × 10^−9^	1.05 × 10^−7^	88.01	*CHRM2*, *GABRQ*, *GRIN3A*, ***CHRNA4***, *CHRM5*, *GRIN2B*
Cholinergic synapse	1.98 × 10^−7^	2.96 × 10^−6^	121.60	*CHRM2*, ***CHRNA4***, *CHRM5*, *CACNA1S*
cAMP signalling pathway	2.64 × 10^−6^	2.97 × 10^−5^	62.19	*CHRM2*, *GRIN3A*, *CACNA1S*, *GRIN2B*
* Arrhythmogenic right ventricular cardiomyopathy	6.46 × 10^−6^	5.81 × 10^−5^	115.34	*CACNB1*, *CACNB4*, *CACNA1S*
* Cardiac muscle contraction	9.33 × 10^−6^	6.64 × 10^−5^	101.56	*CACNB1*, *CACNB4*, *CACNA1S*
* Hypertrophic cardiomyopathy	1.03 × 10^−5^	6.64 × 10^−5^	98.04	*CACNB1*, *CACNB4*, *CACNA1S*
* Dilated cardiomyopathy	1.25 × 10^−5^	7.06 × 10^−5^	91.69	*CACNB1*, *CACNB4*, *CACNA1S*
* Adrenergic signalling in cardiomyocytes	4.77 × 10^−5^	2.32 × 10^−4^	57.85	*CACNB1*, *CACNB4*, *CACNA1S*
Oxytocin signalling pathway	5.16 × 10^−5^	2.32 × 10^−4^	56.31	*CACNB1*, *CACNB4*, *CACNA1S*
Calcium signalling pathway	1.92 × 10^−4^	7.87 × 10^−4^	35.72	*CHRM2*, *CHRM5*, *CACNA1S*
Prion disease	2.81 × 10^−4^	9.73 × 10^−4^	31.30	*GRIN3A*, *CACNA1S*, *GRIN2B*
MAPK signalling pathway	3.50 × 10^−4^	1.12 × 10^−3^	29.01	*CACNB1*, *CACNB4*, *CACNA1S*
Alzheimer disease	6.79 × 10^−4^	1.91 × 10^−3^	22.98	*CHRM5*, *CACNA1S*, *GRIN2B*
Pathways of neurodegeneration	1.41 × 10^−3^	3.34 × 10^−3^	17.72	*CHRM5*, *CACNA1S*, *GRIN2B*

* Non-skeletal muscle results. Bold = candidate migraine target genes.

**Table 4:** Significant pathways derived from the prefrontal cortex gene sublist.

Term	*p*	q	OR	Genes
Calcium signalling pathway	1.36 × 10^−6^	9.27 × 10^−5^	83.71	*CACNA1I*, ***CHRM3***, *CALM1*, *GRIN1*
Circadian entrainment	6.08 × 10^−6^	2.07 × 10^−4^	127.01	*CACNA1I*, *CALM1*, *GRIN1*
* Adrenergic signalling in cardiomyocytes	2.25 × 10^−5^	5.11 × 10^−4^	81.00	*CACNB1*, *CALM1*, *SCN1B*
Neuroactive ligand–receptor interaction	2.58 × 10^−4^	2.77 × 10^−3^	34.89	***CHRM3***, *GABRB1*, *GRIN1*
Alzheimer disease	3.26 × 10^−4^	2.77 × 10^−3^	32.17	***CHRM3***, *CALM1*, *GRIN1*
Pathways of neurodegeneration	6.82 × 10^−4^	3.86 × 10^−3^	24.81	***CHRM3***, *CALM1*, *GRIN1*

* Non-cortical results. Bold = candidate migraine target genes.

## Data Availability

The raw data supporting the conclusions of this article will be made available by the authors on request.

## Supplementary Materials

The following supporting information can be downloaded at: https://www.mdpi.com/article/10.3390/jcm15187040/s1, File S1: Table S1: Compounds perceived as effective for migraine (*n* = 181) arranged by level of evidence found on PubMed. File S2: Table S2: Compounds perceived as effective in migraine and the interacting target genes of interest.
