# Supplementary material for: Integrating Pharmacodynamic Data to Prioritise Candidate Migraine Genes and Pathways Using a Novel Bioinformatics Workflow
Source: J Clin Med. 2026 Sep 11;15(18):7040. doi: 10.3390/jcm15187040 (PMC13607445; doi:10.3390/jcm15187040)
Supplement: Supplementary file 1 [file jcm-15-07040-s001.zip › Supplementary_File_S1.pdf]

Supplementary File S1

Table S1: Compounds perceived as effective for migraine (n=181) arranged by level of evidence found on PubMed.

**Level 1**			
Acetaminophen	Dihydroergotamine	Lisuride	*Rimegepant*
Almotriptan	Eletriptan	Magnesium	Rizatriptan
Amitriptyline	*Eptinezumab*	Mefenamic Acid	Sumatriptan
Aspirin	*Erenumab*	*Melatonin*	Thiamine (Vit B1)
Atenolol	Ergotamine	***Memantine***	Timolol
Atogepant	***Flunarizine***	***Meperidine***	***Topiramate***
Bisoprolol	*Fluoxetine*	Metoclopramide	*Ubrogepant*
Botulinum toxin type A	*Fremanezumab*	Metoprolol	***Valproic Acid***
Butorphanol	Frovatriptan	*Naproxen*	Venlafaxine
Candesartan	*Gabapentin*	Naratriptan	***Verapamil***
Celecoxib	*Galcanezumab*	****Pizotifen***	Vitamin B12
****Chlorpromazine***	*Ibuprofen*	*Prednisolone*	Vitamin B2
Cinnarizine	*Ketorolac*	*Prochlorperazine*	*Zavegepant*
Clonidine	Lasmiditan	Propranolol	Zolmitriptan
Diclofenac	*Lidocaine*	Pyridoxine	
**Level 2**			
Agomelatine	Duloxetine	Lisinopril	Pregabalin
Antipyrine	Estradiol	****Methotrimeprazine***	*Tolfenamic Acid*
Buclizine	*Fenoprofen*	Methysergide	Vitamin D3
Bupivacaine	***Haloperidol***	Nadolol	Vitamin E
Carbamazepine	***Ketamine***	***Nimodipine***	****Zonisamide***
Dexamethasone	*Ketoprofen*	****Nortriptyline***	
Domperidone	*Lacosamide*	*Omega 3 Fatty Acids*	
Droperidol	*Levetiracetam*	Oxygen	
**Level 3**			
Cannabidiol	***Clonazepam***	*Piroxicam*	
Chlordiazepoxide	***Dextropropoxyphene***	*Ropivacaine*	
**Level 4**			
Baclofen	Bromocriptine	****Cyproheptadine***	
**Level 5**			
****Amantadine***	*Dronabinol*	****Methadone***	*Ramelteon*
Caffeine	Ergometrine	*Mianserin*	****Tramadol***
****Cocaine***	Folic Acid (Vit B9)	Oxytocin	Tryptophan
Codeine	*Indomethacin*	Phenelzine	
Dextroamphetamine	Isometheptene	*Prednisone*	
Diazepam	***Lamotrigine***	****Promethazine***	
**Level 6**			
Acebutolol	*Flurbiprofen*	***Lorazepam***	Phenylethylamine
Alprenolol	*Hydrocortisone*	Methylergometrine	Pindolol
Azapropazone	*Hydroxyzine*	****Oxcarbazepine***	Thiethylperazine
****Butalbital***	****Imipramine***	Oxprenolol	Ubidecarenone
Dimenhydrinate	Lithium carbonate	*Phenylbutazone*	
**Excluded**			
Anandamide	Fenclofenac	Lysergic acid diethylamide	Salicylamide
Benorilate	Fosphenytoin	Meclofenamic Acid	Tasimelteon

Cannabis	Ganaxolone	Medical Cannabis	Tenoxicam
Capsazepine	Ginkgolide-B	Methdilazine	Tezampanel
Cyclizine	Indoprofen	Orphenadrine	Tilarginine
Cyclobenzaprine	Ketanserin	Oxitriptan	Tizanidine
Dichloralphenazone	Lithium	Phenothiazine	Tonabersat
Docusate	Lithium cation	Phenytoin	Valnoctamide
Doxycycline	Lithium chloride	Pimozide	
Doxylamine	Lithium citrate	Pirprofen	
Estrogen	Lithium hydroxide	Renzapride	
Ezogabine	Lithium succinate	Rimonabant	

Levels of evidence range from systematic review/meta analyses (1) to animal models/expert opinion (6). *Italics*: interacts with gene list. **Bold**: Interacts with genes of interest. *Interacts with candidate genes.
