# Supplementary material for: Integrating Pharmacodynamic Data to Prioritise Candidate Migraine Genes and Pathways Using a Novel Bioinformatics Workflow
Source: J Clin Med. 2026 Sep 11;15(18):7040. doi: 10.3390/jcm15187040 (PMC13607445; doi:10.3390/jcm15187040)
Supplement: Supplementary file 1 [file jcm-15-07040-s001.zip › Supplementary_File_S2.pdf]

Supplementary File S2

Table S2: Compounds perceived as effective in migraine (n=32) and the interacting target genes of interest (n=21)

Substance Class		Anticonvulsant						Antihistamine				Antipsychotic			Antiviral	Anaesthetic		Analgesic				Barbiturate	Benzodiazepine				CCB			NMDA Antagonist	NSAID	TCA		
**Gene**	**Product**	**Carbamazepine**	**Lamotrigine**	**Oxcarbazepine**	**Topiramate**	**Valproic Acid**	**Zonisamide**	**Cinnarizine**	**Cyproheptadine**	**Pizotifen**	**Promethazine**	**Chlorpromazine**	**Haloperidol**	**Methotrimeprazine**	**Amantadine**	**Cocaine**	**Ketamine**	**Dextropropoxyphene**	**Meperidine**	**Methadone**	**Tramadol**	**Butalbital**	**Chlordiazepoxide**	**Clonazepam**	**Diazepam**	**Lorazepam**	**Flunarizine**	**Nimodipine**	**Verapamil**	**Memantine**	**Diclofenac**	**Imipramine**	**Nortriptyline**	Total
CACNA1G	**Calcium channel**						✓	✓																			✓		✓					4
CACNA1I							✓	✓																			✓		✓					4
CACNA1S					✓			✓																				✓	✓					4
CACNB1					✓																							✓	✓					3
CACNB4					✓																							✓	✓					3
CALM1	**Calmodulin**										✓	✓															✓							3
CHRM2	**Acetlycholine receptor**								✓	✓	✓			✓		✓																✓	✓	7
**CHRM3*									✓	✓	✓	✓		✓							✓											✓	✓	8
CHRM5											✓			✓																		✓	✓	4
**CHRNA4*															✓					✓		✓												3
GABRA6	**GABA receptor**																					✓	✓	✓	✓	✓								5
GABRB1																						✓	✓	✓	✓	✓								5
GABRD																						✓	✓	✓	✓	✓								5
GABRQ																						✓	✓	✓	✓	✓								5
GRIN1	**Glutamate receptor**																	✓	✓	✓	✓									✓				5
GRIN2B													✓					✓	✓	✓	✓									✓				6
GRIN3A															✓		✓	✓		✓	✓									✓				6
SCN1A	**Sodium channel**	✓	✓	✓	✓	✓	✓				✓					✓																		8
SCN1B				✓			✓									✓																		3
**SCN2B*				✓			✓									✓																		3
SCN4A		✓	✓	✓	✓	✓	✓				✓					✓															✓			9

CCB = Calcium Channel Blocker, TCA = Tricyclic Antidepressant, * Candidate migraine target genes
